# Animal-Assisted Interventions in the Classroom—A Systematic Review

**DOI:** 10.3390/ijerph14070669

**Published:** 2017-06-22

**Authors:** Victoria L. Brelsford, Kerstin Meints, Nancy R. Gee, Karen Pfeffer

**Affiliations:** 1 School of Psychology, University of Lincoln, Brayford Pool, Lincoln, Lincolnshire LN6 7TS, UK; vbrelsford@lincoln.ac.uk (V.L.B.); KPeffer@lincoln.ac.uk (K.P.); 2 Department of Psychology, State University of New York, Fredonia, NY 14063, USA; nancy.gee@fredonia.edu; 3 WALTHAM™ Centre for Pet Nutrition, Waltham-on-the-Wolds, Melton Mowbray, Leicstershire LE14 4RT, UK

**Keywords:** animal-assisted intervention, dog, classroom, school, children, learning

## Abstract

The inclusion of animals in educational practice is becoming increasingly popular, but it is unclear how solid the evidence for this type of intervention is. The aim of this systematic review is to scrutinise the empirical research literature relating to animal-assisted interventions conducted in educational settings. The review included 25 papers; 21 from peer-reviewed journals and 4 obtained using grey literature databases. Most studies reported significant benefits of animal-assisted interventions in the school setting. Despite this, studies vary greatly in methods and design, in intervention types, measures, and sample sizes, and in the length of time exposed to an animal. Furthermore, a worrying lack of reference to risk assessment and animal welfare must be highlighted. Taken together, the results of this review show promising findings and emerging evidence suggestive of potential benefits related to animals in school settings. The review also indicates the need for a larger and more robust evidence base driven by thorough and strict protocols. The review further emphasises the need for safeguarding for all involved—welfare and safety are paramount.

## 1. Introduction

The use of animals as part of educational and therapeutic interventions for humans has greatly increased over recent years with many types of animals such as dogs, guinea pigs, rabbits, horses, and even farm animals included within educational settings and therapeutic programs. Benefits of animal-assisted interactions (AAI) for both psychological and physiological health of humans in a variety of environments have been highlighted [1,2,3,4].

Health benefits of companion animals have been observed in various domains within human health and wellbeing. For example, early research by Friedmann, Katcher, Lynch, and Thomas [5] linked pet ownership to improved cardiovascular health and demonstrated it to be a significant predictor of survival in patients one year after heart attack. Many studies have since linked AAI with positive health outcomes, for example in pain management, in the treatment of depression, and in wider neurological rehabilitation studies (for an overview, see Munoz Lasa et al. 2015) [6]. For example, children have reported a reduction in pain in the presence of an animal, and positive effects have been observed in children and adolescents hospitalised for acute mental disorders, reducing emotional and behavioural symptoms and increasing global competence and psychological functioning [7,8,9]. Elderly patients also benefit from animal interactions to combat depressive symptoms, and positive effects such as improving and enhancing socialization among older adults with dementia in care homes are reported, resulting in fewer incidents requiring staff intervention [10,11].

Animal intervention has also been linked to positive behavioral improvements in teenagers with acute mental disorders such as mood disorder, schizophrenia, anxiety, and eating disorders—significant improvements occurred through interventions with a dog [12]. Furthermore, youths in residential care displayed more secure attachment following animal-assisted therapy [13]. Indeed, companion animals have been found to influence children’s development positively as well as demonstrate positive benefits across the lifespan [2,14].

Research investigating the effects of human–animal interactions (HAI) on physiological measures of wellbeing often demonstrates positive results. Increases in oxytocin production and decreases in cortisol levels, blood pressure, and galvanic skin measurements all point to a clear relationship between HAI and the reduction of stress and anxiety in humans. In this respect, animals may act as social buffers to the impact of psychosocial stress, by ameliorating its impact in humans, as well as providing a wider therapeutic role in human wellbeing [15,16]. It also appears that this may be a two-way interaction with mutual benefits in both dogs and humans [17].

More recently, the application of pets in the classroom as beneficial aids to learning has become more popular, but it is unclear how robust the evidence for such classroom-based interventions is. For example, Austria has changed their teaching practice in schools after having developed guidelines that enable teachers to bring their pet dogs into schools [18,19]—for a detailed overview, see Gee [19]. A survey with 1400 teachers showed that almost 70% employed animals in the classroom, with advantages found in attention, motivation, mood and wellbeing, socio-emotional development, and empathy, as well as cognitive development [2,20,21,22,23,24,25,26,27,28] (for a detailed overview of animals in educational settings, see Gee [1]). However, whilst current literature demonstrates a wide variety of anecdotal evidence in relation to the benefits of animals in educational settings and research publications into wider HAI and AAI are increasing steadily, methodological approaches and timeframes used across environments and treatments often vary and lack rigorous experimental designs making it difficult to draw conclusions [29]. This makes the comparison of potential beneficial effects across studies very difficult. The aim of this review is to scrutinise the empirical research literature relating to animal-assisted interventions conducted in educational settings. In order to judge the scope and validity of the evidence, a systematic review of the research was conducted and can now be used to inform and improve future investigations.

So far, previous systematic literature reviews have been conducted examining the impact of AAI on child and adolescent health and wellbeing [30,31,32,33,34]. Some focused on the effect of animals on children and adolescents with Autism Spectrum Disorder (ASD) [35,36], and one review focused on children who read to dogs [37]. Overall, reviews have consistently reported the beneficial effects of HAI across the research they evaluate. Conversely, many shortcomings in these research papers have also been reported. For example, O’Haire [36] reported a positive increase in the number of AAI studies relating to children with ASD, with studies being variable and spanning a variety of disciplines. However, the review also highlighted a lack of consistent terminology and research protocols, and revealed that studies were often poorly described. O’Haire [36] concluded a need for more rigorous research and a streamlining of the terminology to unify future research. Davies et al. [35] also assessed the AAI literature with a focus on changes associated with ASD symptoms (i.e., social communication and stereotyped or challenging behaviour). They concluded that a positive relationship between children and animals exists, but criticised a lack of efficacy of the interventions and multiple methodological flaws across the literature under review. Likewise, Kamioka et al. [32] reviewed research relating to animal-assisted therapy focusing on randomised controlled trials (RCTs) and highlighted ‘serious problems with the conduct and reporting of the target studies’ (p. 385), with many of the studies being of relatively low quality. It is therefore imperative that researchers take note of these criticisms to ensure that future studies are designed and carried out to a high standard, i.e., that methodological flaws are eradicated, and protocols are consistent and appropriate. Hall et al. provided insight into studies on reading with dogs, concluding that dogs may have a beneficial effect on several behavioural processes, but much of the evidence on which these assertions were made is of low quality and lacks the inclusion of standardised measures [37].

**Figure 1:**
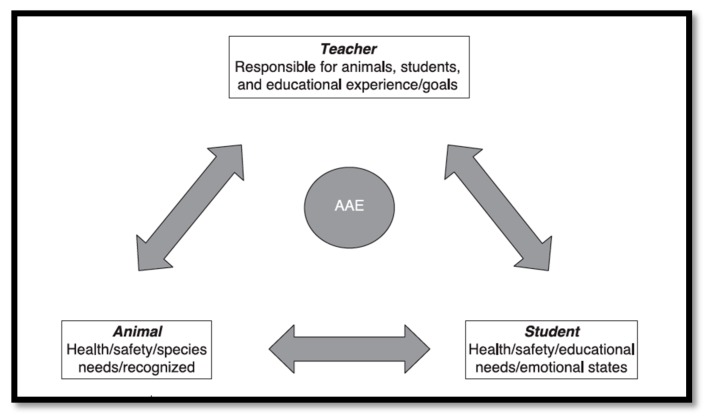
Reprinted from Handbook of Animal-Assisted Therapy, 4th ed.; Gee, N.R.; Fine, A. & Schuck, S., Animals in educational settings: Research and practice, pp. 195–210, 2015, with permission from Elsevier [19].

Thodberg, Berget, and Lidfors [38] and Gee et al. [19] provide a series of recommendations for research involving the use of animals as a treatment for humans. Thodberg et al. [38] suggested that to fill our existing knowledge gap in relation to AAI, the field requires a consistent approach across multi-disciplinary areas. The effectiveness and measurement of interventions must be clear and appropriate, with control groups used as an important feature of study design. They also called for the collection of longitudinal data to help ascertain both direct and underlying pathways between the factors involved, and stressed the importance of observational and physiological data as essential components in measuring the quality of interactions and their effect on humans. Lastly, but crucially, the needs and welfare of animals taking part in animal-assisted interventions need to be fully met. Gee et al. made similar suggestions, and added a useful working model of how interventions can be successfully managed and monitored between pupil/student, animal, and teacher (see Figure 1) [19].

Due to the growing popularity and demand for the use of animals, especially in the classroom, and due to the lack of best practice recommendations for empirical research, the current review differs from the above reviews in that it focuses specifically on animal-assisted interventions that have been carried out within the classroom. Animal-assisted interventions are used in schools with typically developing children and are often employed with children with reading difficulties. Such interventions are also used frequently in schools for children with special educational needs, for example, with children with ASD and other disabilities (see search terms below).

To build a coherent picture of the usefulness of current animal interventions within educational settings, this review will summarise and evaluate existing research and its outcomes in all types of schools. We will compare specific details of the studies, and findings will be discussed with a view to inform and improve future research efforts. Finally, we will make recommendations for research and working practice with animals in the classroom.

## 2. Materials and Methods

This systematic literature review is reported in accordance with PRISMA guidelines (Preferred Reporting Items for Systematic Reviews and Meta-Analyses) and the PRISMA checklist [39]. PRISMA provides an evidence-based minimum set of items for reporting in systematic reviews through a 27-item checklist.

Search methods, eligibility, and exclusion criteria were specified in advance. The following eligibility criteria were applied:

(A) All studies had to be conducted in formal educational settings involving children and adolescents from 2;6 to 18;0 years. This incorporates formal early years’ settings and schools. Studies were eligible if they
  (a) incorporated a real animal within the study design; and
  (b) reported any cohort size, including case studies.
(B) Only studies reporting empirical research were used, including experimental, cross-sectional, longitudinal, randomized controlled, and case study designs.
(C) Only research published in peer-reviewed journals was included.

All types of empirical research were included as to inform about all research conducted within classroom settings and to avoid omitting relevant research. Dissertations and post-graduate theses were included as part of a grey literature search. As these were not necessarily published, they would not meet criteria ‘C’ above; in all other respects, they were expected to meet the eligibility criteria to be included in the review.

Nine databases were searched from their start date to present for peer-reviewed articles. These included Academic Search Complete (1965–present), Child Development & Adolescent Studies (1927–present), Frontiers in Science (2002–present), Medline (1946–present), PyschArticles (1894–present), PsychInfo (1967–present), Science Direct (1946–present), Scopus (2005–present), Taylor & Francis online (including Anthrozoös, Childhood Education and Educational Review Journals) (1990–present), and Web of Science (including Web of Knowledge) (1970–present). Two databases were also searched for grey literature: ProQuest Dissertations & Theses Global and the System for Information on Grey Literature in Europe (http://www.opengrey.eu/).

Additional literature was searched using three websites dedicated to the study of human–animal interactions: WALTHAM Science (https://www.waltham.com/waltham-research/ hai-research/hai-resources/), HABRI-Central (https://habricentral.org/resources/) and Animals and Society Institute (https://www.animalsandsociety.org/human-animal-studies/society-and-animals-journal/).

Search terms ‘animal-assisted intervention’, ‘canine-assisted intervention’, and ‘dog-assisted intervention’ were combined with search terms ‘children’, ‘education’, ‘school’, ‘classroom’, ‘learning’, ‘autism’, or ‘reading’, all words were combined with all search terms to return the maximum amount of potentially relevant results.

The first author conducted an initial search of databases in February 2016. A further search was conducted in February 2017 to ensure all new material was included within the review. Titles were scrutinised to ensure they related directly to the topic of the review. Articles were then screened through the abstract to ensure they fitted the eligibility criteria. Papers deemed valid to the review were then systematically searched to obtain specific information required for the analysis to take place. In addition to authorship information, further information was extracted in relation to demographics (cohort size, age, gender, characteristics such as learning disabilities, formal diagnoses), methodology (control type, type of animal, experimental tasks, timing of intervention), measures collected (e.g., cognitive, behavioural, physiological, ethological), and animal welfare and ethical issues (length of animal contact time, training level/certification of dogs, allergy information, risk assessment, and ethics).

## 3. Results

**Figure 2:**
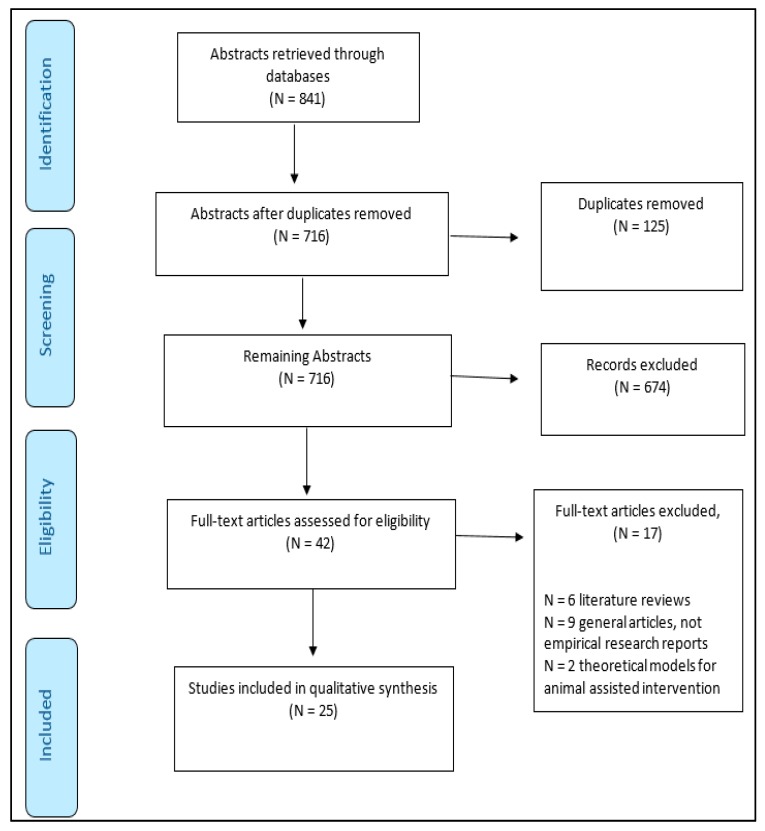
PRISMA (Preferred Reporting Items for Systematic Reviews and Meta-Analyses) flow chart.

The search returned 841 articles. A large proportion of the articles excluded at this stage were related to assisted education and educational interventions, but did not include animals within the classroom. Many of the published articles returned in the search did include animal interventions, but did not necessarily take place within the educational setting and were also excluded. This left 167 remaining papers, and duplicates were removed (N = 125), leaving 42 research papers for review. The remaining 42 articles were subjected to additional scrutiny to ensure they fit the eligibility criteria. A further 17 papers were removed: six were not included due to being systematic literature reviews, nine were general articles related to animal-assisted therapy rather than empirical research publications, and two were related to theoretical models of the application of animal-assisted therapy. There were 25 eligible articles remaining for review (see Figure 2).

The Oxford Centre for Evidence-Based Medicine (OCEBM) Levels of Evidence (2011) [39] were applied to the remaining articles (see Table 1 and Table 2). Articles were grouped by first and second authors independently and agreement reached on any differences. N.B.: The levels are not intended to provide a definitive judgment on the quality of evidence (see OCEBM Introductory document) [40].

The 25 articles are reports of empirical research carried out with children and adolescents in educational settings and involved three different animals: three involved the use of guinea pigs, one included a rabbit, and the remaining twenty-one included the assistance of a dog. Of these, 16 studies were related to research carried out with school-aged children in an educational environment, and 8 were related to research carried out with pre-school (or kindergarten) children in nursery settings, and 1 spanned both age ranges.

Studies included intervention with typically developing children, in addition to emotional and behavioural disabilities, Downs Syndrome, Autism Spectrum Disorder, including Asperger’s Syndrome, oppositional defiance disorder, attention deficit disorder with hyperactivity, reactive attachment disorder, intermittent explosive disorder, central auditory processing disorder, visual processing challenge, auditory processing challenge, and attention focus challenge listed as diagnoses within the publications.

The focus of investigation across many of the studies was largely socio-emotional, including the effect of animals on mood, emotional regulation, and social behaviours and functioning. Other topic areas included the effect of animals in the classroom on the following: insecurely/disorganised attachment behaviours, reading rate, accuracy and comprehension, adherence to instructions and instructional prompts, categorisation and object recognition, motor tasks, and lastly general classroom behaviour.

To facilitate discussion of the papers in this review, the studies are grouped and reported based on the topic of investigation in the first instance, and will be followed by a wider discussion, which presents a critical overview of the research as a conclusion to the review. Statistics will be presented where possible depending on whether they were reported in the articles.

### 3.1. Reading Ability

The search returned four relevant studies that met our criteria. These four studies involved the use of reading programs combined with canine intervention in kindergarten and school-aged children. All studies were carried out in educational settings; however, because they included distinctly different populations of children and differing study designs, direct comparison of the outcomes across these studies is difficult. Bassette and Taber-Doughty [42] used a single case design of pupils (N = 3) with emotional and behavioural difficulties, aged 7 years (N = 1) and 11 years (N = 2) and assessed ‘on task behaviours’ through a multiple probe technique. Le Roux, Swartz, and Swartz [53] included typically developing children who had been identified as poor readers and were between the ages of 7–13 years (N = 102) in their randomized controlled trial, which assessed the effect of the presence of a dog on reading rate, accuracy, and comprehension. In contrast, both Kirnan et al. [50] and Treat [58] included larger cohorts whose ages spanned across a range of year groups. Kirnan et al. [50] investigated a cohort that ranged from kindergarten to Grade 4 pupils (9–10 years, N = 169), some in ‘traditional classrooms’, others in special education classrooms (although numbers are not specified). Treat’s [58] study involved children with specific learning disabilities and spanned across year groups from Grades 2 to 5 (7–11 years). Children took part in guided reading aloud sessions with or without a dog.

Bassette and Taber-Doughty [42] investigated three pupils’ educational engagement whilst reading in the presence of a dog. On-task behaviour was measured using Interval Recording [60], a type of Applied Behavioural Analysis (ABA) observational record of whether a behaviour occurs during intervals of a specified time period. The accelerated reader (AR) quiz program (an internal daily school reading assessment) was used to assess reading comprehension. Each child read individually for half an hour per morning in the presence of a dog for a period of 4 weeks. Children were assessed on their reading comprehension and on-task reading aloud behaviour prior to and following intervention. After one month, children were assessed on the maintenance of on-task behaviours without a dog present. The authors presented the percent of intervals on-task reading aloud and stated a moderate to significant improvement of ‘on-task’ behaviours. Despite using the AR program, results were not presented in the result section and seem inconclusive. Teachers also completed a Social Validity Interview and reported improvements in behaviour during intervention. No standardised measures were completed as part of the evaluation. Given the above weaknesses in assessment and analysis, it is hard to draw conclusions from this study.

Le Roux et al. [53] evaluated the effect of the presence of a dog on children’s reading ability using a pre–post test design with a control group. They addressed the potential confounders of using single dogs and single handlers by employing several dogs and several handlers. All dogs were trained therapy dogs, all dog handlers were trained and received additional training on the task. Familiarisation was carried out for all groups before testing. For assessment, the Reading Educational Assistance Dogs program (READ) was employed, and three further conditions were included: reading to a human, reading to a teddy bear, and a control group with no intervention. Children were randomly assigned into test groups and read to the human, dog, or teddy bear for 20 min per week over a 10-week intervention period. Reading was assessed using the Neale Analysis of Reading Ability before the intervention, directly after 10 weeks and again eight weeks later. The main effect for the group was significant (*F*(3, 94) = 3.40, *p* = 0.2, η^2^ = 0.9) with the dog group (*M* = 7.94, *SD* = 0.96), demonstrating a higher reading rate than the teddy bear group (*M* = 7.45, *SD* = 7.9) and significantly higher reading accuracy than all other groups. A significant main effect was also found for reading comprehension age scores (*F*(3, 94) = 5.02, *p* = 0.02, η^2^ = 0.15) with the dog group, showing significantly higher comprehension scores than all other groups. The authors reported that eight weeks after the completion of the reading program, students in the dog group retained their lead over the students in the other three groups. However, reading comprehension was the main ability retained by students in the dog group; not all reading skills were maintained after the 10-week intervention. Both Bassette and Taber-Doughty [42] and le Roux et al. [53] concluded that animal intervention has a role to play in academic engagement and reading skill. Both studies also acknowledged that further work is needed to tease apart the factors involved; for example, immediate improvement in measures followed by high variability may be due to initial motivational factors. The presence of a dog may improve reading by affecting physiological measures such as blood pressure. It is also possible that effects are due to the dog or the individual present during intervention, including the bonding process.

Kirnan et al. [50] assessed the effect of a therapy dog on the reading skills of children across a whole school cohort from Kindergarten to Grade 4. Children in ‘traditional’ classes typically read to the dog in groups of 4–6, whilst children in special education classes read to the dogs individually; dogs attended each class for approximately one hour per week. Children also took part in a writing component such as writing dog themed articles or journals. Children in kindergarten and Grade 1 experienced a more integrated dog program through a language arts curriculum that included writing about dogs, illustrating writing, and playing vocabulary games based around a dog theme. Reading scores were assessed mid-year (Winter scores) prior to intervention, and at year-end (Spring scores) through school Measures of Academic Progress (MAP). MAP scores from a previous cohort of children (2010/11) were used as a control measure to compare reading scores to those in the dog group (2011/12). The analyses found a statistically significant difference in kindergarten with the dog reading group ending the year, with significantly higher reading scores compared with the control group (control (*M* = 160.34, *SD* = 11.97); dog (*M* = 169.96, *SD* = 10.04), *t* = 3.35, *p* > 0.001). No other year group demonstrated significant differences in reading scores between dog and control conditions. The authors acknowledged that using the previous academic years’ performance as a control is problematic, and reading score gains could be influenced by cohort differences or historical events. Indeed, factors such as classroom environment, the effect of a different teacher, and the dynamics of the class as a whole cannot be controlled for, so their impact is unknown. There is also an assumption on the part of the researcher that all year groups are performing at the same level year on year; in reality, this may not be the case, certain year groups of children may perform worse or better than others.

Treat [58] assessed the effect of a certified therapy dog on the reading abilities of 17 children (male N = 11, female N = 6) who had identified learning disabilities, stated as a visual processing challenge, auditory processing challenge, and attention focus challenge. The intervention involved each child taking part in guided reading aloud with a teacher/researcher. Children were assigned to either a dog or no-dog group. Reading sessions lasted 10–15 min (depending on story length) and took place 1–3 times per week depending on the availability of the child. Each child had 10 sessions of intervention. Interventions also involved the child receiving instruction and feedback on strategies to assist with their reading comprehension and fluency during the sessions. Only the dog group was assessed pre- and post-intervention using the Gray Oral Reading Test (GORT-4). Both groups were assessed pre- and post-intervention using the Basic Reading Inventory (BRI) and the Reader Self-Perception Scale (RSPS). The latter was used to gauge perceived self-efficacy in reading upon completion of the intervention. An anxiety measure was also completed, although this was a questionnaire devised by the researcher and not a standardised tool. The study demonstrated significant improvement between pre- and post-test scores on the GORT-4 for reading rate, accuracy, fluency, comprehension, and reading quotient in the dog group. The comparison group was not subjected to the GORT-4 pre-post assessment due to a lack of time, leaving open the specific effect of the dog during the reading sessions. The other two measures showed improvement before and after intervention; however, only descriptive data is provided, and no statistical analysis was employed, so it is unclear if the differences are statistically or clinically significant.

The study acknowledged, but failed to control for the fact that some of the children had already been receiving part of the intervention strategy implemented by the teacher for a year before the study commenced. This is problematic, as it remains unclear if any of the observed effects were due to the intervention applied during the research period. Further analysis of those children who had already been receiving part of the intervention strategy could have previously clarified this issue and answered this question.

### 3.2. Emotional Stability and Learning

Four of the papers within the review focused on the improvement of the socio-emotional wellbeing of pupils, all involved the presence of a dog and all looked at the impact of the outcomes on the potential to support learning in the classroom environment. Two of the studies used a case study design [41,51] and two involved a larger group of children [46,47].

Anderson and Olson [41] used a case study design to investigate the effect of the presence of a dog on the emotional stability and learning of students with a range of severe emotional disabilities, including oppositional defiant disorder, attention deficit disorder with hyperactivity, reactive attachment disorder, intermittent explosive disorder, central auditory processing disorder, mood disorder, bipolar disorder, and Asperger’s Syndrome. The sample included six children aged 6–11 years who had been unsuccessful in the mainstream school environment and who were placed in a self-contained classroom accompanied by one-to-one tutors. Children were observed and their behaviour documented for 8 weeks, and the intervention took place over an 8-week period with the dog present in class each day between 8 a.m. and 3 p.m. with the same observation and documentation. Each child took part in one-to-one sessions for 30 min every day. Children also interacted with the dog throughout the school day, socialising with the dog during educational activities such as reading and playing with the dog during break-times. The dog was not a trained therapy dog and had limited experience with children, but the authors put risk-reducing strategies in place. Both qualitative and quantitative data were collected, including parent and teacher reports before and after intervention through Problem Solving Sheets and ABC analysis forms. ABC Problem Solving Sheets were completed when the child entered an emotional crisis. Sheets allowed the recording of Antecedents (A), events that preceded the crisis, Behaviour (B), observed behaviour, and Consequences (C), consequences and events following the crisis. No standardised measures were collected. No separate control group was tested. The authors collected observational data, interview data with the children and their parent. They used qualitative analysis and reported that the dog contributed to the children’s overall emotional stability, improved behavioural control, and students’ attitudes towards school and facilitated the students learning in relation to responsibility, respect, and empathy. Overall, the dogs had positive socio-emotional effects on the students. The presence of the dog in class for the entire 8-week period during the day makes it difficult to discern the potential advantage to the children of the one-to-one sessions with the dog and demonstrate whether the mere presence of the dog in the classroom without personal intervention with each child may still have had the same effect. A further concern relates to the potential bias in interpretation of the results obtained by the teacher/researcher. The authors acknowledge that ‘the lack of negative findings’ (p. 47) could be questioned as an effect of teacher expectations; a comparison condition within the design of the study would have controlled for this potential bias. 

Kogan et al. [51] also used a case study design with two male children. Child A was 11 years old and was diagnosed as having mild intellectual disability disorder, attention deficit disorder, oppositional defiant disorder, depression, and explosive tendencies. Child B was 12 years old and described as hyperactive and having depression and problems with impulse control. Both children were identified as emotionally disturbed. Each child took part in weekly sessions of between 45 and 60 min with a therapy dog. Sessions consisted of two parts: rapport-building time and animal time. Children could interact with the dog, including activities such as petting or brushing the dog as well as discussing previous positive and negative events with the handler. Goals were set by the Special Educational Needs teacher to work on during the AAI sessions. Standardised Teacher Social Skills rating scales were completed before and after intervention. Individual Education Plans were assessed, all sessions were videotaped, and post-intervention interviews with children, families, and educational professionals were carried out. Positive results were reported for both case studies, with Child A demonstrating a reduction in negative verbal statements and increased positive ones. These positive communication skills transferred into daily activities during interaction with others, such as an improved voice tone and increased patience when dealing with peers. Child B showed noticeable improvements across learned helplessness, with his sense of control of himself and his environment improving dramatically. Working closely with the dog was reported to have had a direct influence on their sense of control by decreasing feelings of helplessness and improving self-confidence. The study focused on goal-setting and regulation of behaviours during intervention, with the authors asserting that both children improved on most of their goals after intervention with a dog. It is difficult to assess the direct beneficial effect of the dog alone as the intervention consisted of a human–dog team.

In contrast, Beetz [46] assessed the intervention of a ‘school dog-teacher-team’ together on the social interactions of children within the classroom. The study consisted of children between the ages of 8 and 9 years (N = 46) with equal numbers of both males and females (N = 23) (dog cohort age: *M* = 8.5, *SD* = 0.51; control cohort age: *M* = 8.4, *SD* = 0.51). Two cohorts were used: one classroom was visited by a dog for one day per week over the course of a year, and another class with no dog served as the control. The study involved a comprehensive battery of standardised questionnaires assessing the socio-emotional wellbeing of the children before the intervention began and again following intervention. Depression scores did not differ across time as a result of intervention in either the dog or control condition, but positive attitude towards school and positive emotions towards learning, as measured by the FEESS 3-4 (Questionnaire on Emotional and Social experiences in school, Grades 3–4), rated significantly higher in the dog group than the control group with no dogs. The researchers attributed a medium to strong benefit to the whole class as a group. Worryingly, the no-dog group demonstrated a decrease in their attitudes towards school and emotions towards learning. The authors pointed out that the third year for students in Germany is a time of high academic pressure and that the dog may have acted as a buffer during this stressful period in which the control class did not have access to a dog.

This could add to the argument of the beneficial effect of a dog in the classroom; however, it is also possible that the children in the no-dog class were aware that the other classroom had a dog and that their classroom did not. This awareness could be responsible for the declining attitudes towards school and emotions towards learning found in the no-dog group.

Lastly, Donaldson [47] investigated the use of a therapy dog to promote empathy in pre-school classes. The study involved N = 47 children aged 3;8–4;11 years (male (N = 23), female (N = 24)), and a PAWS trained therapy dog and their handler were involved. Three classes were used as condition groups (no random assignment); one with a therapy dog, one with a plush toy dog, and one control group with no additional adjunct. It is important to note that, as in the above studies, intervention is confounded with classroom/teacher/students.

Children were assessed using the Emotion Matching Task (EMT), and two scores from the Strengths and Difficulties Questionnaire (SDQ). Video footage was also collected in the dramatic play area, this was analysed for prosocial, aggressive, and isolation behaviours. Children in the toy and real dog conditions did not improve in emotional recognition over the course of the intervention. Equally, prosocial and difficulty scores from the SDQ failed to demonstrate significant improvement. While some of the qualitative data suggested improvements in children’s behaviour, no discernible differences were observed in relation to prosocial, aggressive, or isolation behaviours coded from the video data. While the previous three studies reported positive emotional effects and the potential of animal-assisted intervention to support learning and educational goals, Donaldson [47] found no significant effects of the dog condition over others. Importantly, the researchers concluded that the factors involved need quantifying, with two of the papers acknowledging confounding factors, i.e., the difficulty in controlling for wider factors, such as teachers, classroom environment, and other intervention programs in which individual children may already be participating [41,46].

### 3.3. Social Functioning and Interpersonal Skills

Four of the studies identified in the review investigated the effect of animals on social functioning in the classroom. O’Haire et al. [55,56] included guinea pigs as the animal of choice in their studies, whilst Tissen et al. [57] and Wicker [59] involved dogs in their studies. 

The first two studies with guinea pigs in the classroom environment aimed to investigate the social functioning of children [55,56]. Importantly, the authors highlighted that the aim of the research was to examine the impact of the intervention on the functioning of the children within the classroom, as opposed to the specific role of the animal involved.

Their study constitutes two papers; one reported the results of the study with typically developing children (N = 128) aged 4.8–12.7 years [55], and the other [56] reported the results of the effect on children with Autism Spectrum Disorder (N = 64) aged 5.2–12.8 years old. Guinea pigs were placed in classroom settings and the children assigned to either an Animal-Assisted Activity (AAA) group or a wait list control group. All children received general exposure to the guinea pigs in the classroom setting, the experimental group received in addition separate AAA interaction sessions. AAA sessions were carried out in triads with two typically developing children, and one with ASD taking part in intervention activities for 20 min, twice per week over an 8-week period. The activities carried out during the intervention sessions were led by pupil preferences and included a wide range of activities such as feeding, designing experiments, grooming, visual art, and circle time. Standardised tests were used to assess the children’s behaviour. These included the Pervasive Developmental Disorder Behaviour Inventory (PDDBI) and the Social Skills Rating Scale (SSRS), which included parent and teacher assessments of whether children showed changes in their interest in attending school. Information relating to the child’s school, teacher, academic grade, pet ownership, and outside treatment were also collected.

The authors reported that pupils with ASD displayed more social approach behaviours and less social withdrawal following AAA as reported by both teachers (*p* < 0.001) and parents (*p* < 0.007). Participants were also rated as more socially skilled by teachers (*p* < 0.008) and parents (*p* < 0.006). However, problem behaviours showed no improvement following AAA [56]. Typically developing pupils were also reported to have benefitted from the AAA, with pupils showing significantly greater increases in social skills as rated by teachers (*p* < 0.001) and significantly greater decreases in problem behaviours as rated by both teachers (*p* < 0.001) and parents (*p* = 0.003). Both studies included standardised measures; however, both these measures rely on feedback from teachers and parents who were not blind to the conditions, so results are open to expectation bias. Academic competence of the typically developing children was also rated by teachers through the SSRS [55]. The study used a wait list control condition and whilst this is often rated as superior to a no control design, it is not without drawbacks. It is impossible to know whether the wait list condition was potentially negatively affected by motivational factors and an alternative control group may be more useful. 

Tissen et al. [57] investigated social behaviour, empathy, and aggression in their study with children (N = 230) aged 7–10 years old. They investigated the effect of social training programs using six therapy dogs and their handlers. Children’s school classes were randomly assigned to conditions as follows: social training without dogs, social training with dogs, and dog attendance without social training. They implemented a 10-week program with 90-min of intervention per week in the classroom setting. Standardised questionnaires were carried out with teachers and children before and after interventions and again 3 weeks later with children only. These include the Social Behaviour Scale: Assessment aids for teachers, the Inventory for the Assessment of Impulsivity, Risk Behaviour and Empathy (for children), and a Bully/Victim-Questionnaire for children. The authors reported a minor significant effect in social behaviour from the teachers’ perspective after the 10-week intervention compared with the start (*p* < 0.05). Measures of empathy also showed a ‘low but significant’ effect (*p* < 0.05). Neither of these were not maintained beyond the intervention. The authors found only a reduction in the condition ‘Social training with dogs’ in open and relational aggression and effects lasted beyond the intervention time. No main effects for condition were found. Hence, it would therefore appear that the presence of a dog can potentially have the same effect as social training and vice versa. Unfortunately, the lack of a no-intervention control does not allow the results to be assessed further, as all children were either exposed to a dog or social training. The authors acknowledged this and suggested further improvements to their study. 

Wicker [59] investigated the effects of animal-assisted therapy with dogs on the social behaviour and interpersonal skills of N = 31 at-risk adolescents aged 12;2 to 17;5 years (female, N = 9, male N = 22) attending alternative public schools. Two of the 6 children were classed as having emotional difficulty and one ‘ASD-like’ (p. 38) behaviour. Students in the intervention group (N = 20) had an intervention incorporated into their educational plan. Control students (N = 11) were located on a different campus because not enough children consented to take part at the first school. Social skills, aggressive behaviour, attitude to school, interpersonal relations, classroom absences, direction following, acceptance of staff feedback, and respectful responses were measured using a standardized tool (BASC). Students were not randomly assigned to each condition, but were assigned by staff members who saw the child in ‘critical need of a more intense intervention’ (p. 38) into one-to-one sessions for one hour per week. Others were allocated to small groups of five students twice per week for one hour with the dog.

Students were instructed on how to train and care for the dog. A certified dog trainer was present to oversee sessions. The dogs belonged to members of the community (not the trainer). At the end of the intervention, students demonstrated their dog handling and training skills to the dog owners. The study failed to find any significant effects between the three cohorts in terms of teacher ratings of students’ social skills, aggressive behaviour, attitude to school, interpersonal relations and classroom absences, and the authors acknowledged the limitations of their small sample size for analysis. The study provided an insight into the personal experience of those taking part through qualitative information gathered after intervention.

In sum, three of the studies reported benefits of animal intervention in their findings, but equally all three outlined the protocol for control group as an area for improvement in future studies to ensure that outcomes can be directly attributable to the intervention, rather than wider factors. Further to this, the importance of gathering more data around participant characteristics such as ability and diagnoses as well as questions concerning scale and amount of interventions will allow researchers in the future to analyse how these individual differences may mediate the intervention under review.

### 3.4. Physiological Arousal

Four studies in the review focused on the physiological factors involved during children’s interactions with animals in intervention sessions [15,43,44,45].

Beetz [44] carried out an exploratory study involving male children (N = 31) between the ages of 7 and 12 years old, investigating the effects of a real dog, toy dog, or friendly person on insecurely/disorganised attached children during a stressful task. Having chosen participants on the basis of preselection using the Separation Anxiety Test (SAT), a series of standardised tests were completed, the Trier Social Stress Test for Children (TSST-C) in combination with Self-Assessment of Stress, The Self-Assessment Manikin (SAM), and a pet attachment questionnaire. Salivary cortisol was taken at five points throughout the intervention process (T1 to T5). No difference between the three groups was initially found in relation to pet attachment (F = 0.743, *p* = 0.537). No significant differences were found for self-reported Activation and Mood (SAM) during or after intervention. Self-reported stress levels did not differ significantly between the groups before or after the stress test (TSST-C), but cortisol analysis revealed a significantly lower level in the real dog condition (x^2^ = 15.17, df = 2, *p* = 0.001) (measured under the curve (AUCi). This was also related to the amount of time stroking the dog; more direct contact time resulted in a less pronounced reaction to stress as cortisol was lower at T5 (r_s_ = −0.818, *p* = 0.002). 

Further to this, Beetz [45] investigated the effects of social support by a dog on stress modulation in male children with insecure attachment (N = 47) aged 7–11 years. Measures were collected as in the previous study with similar findings that male children with insecure-avoidant/disorganised attachment can profit from the presence of a real dog with cortisol levels at T4 and T5 being significantly lower (x^2^ = 6.17, df = 2, *p* = 0.046) (measured under the curve (AUCi)) correlating negatively with the amount of physical contact with a real dog (increased stroking resulted in decreased cortisol readings). The more time the child spent stroking the dog before the TSST-C stress test, the greater the drop in cortisol level (r_s_ = 0.488, *p* = 0.025). Again, children’s self-reported stress did not correspond with salivary cortisol levels. 

O’Haire [15] evaluated physiological arousal as a mechanism for observed behavioural changes in the presence of guinea pigs. The cohort (N = 114) consisted of both typically developing children (TD) (N = 76) and children with ASD (N = 36) with a wide age range from 5.1 to 12.7 years old. The study collected data using a series of standardised questionnaires including the Social Communication Questionnaire (SCQ) (Autism screening), the Social Skills Rating System (SSRS) (social functioning), and the Social Worries Questionnaire (SWQ). In addition, parent and teacher’s ratings of the character of each child were collected and emotional valence rated by children about how they felt after taking part in each condition. The study also collected skin conductance measures, including temperature and motor movement data. Children took part in all four experimental conditions in the same order: baseline reading silently, reading aloud in front of peers for 1 min, 10 min of free play with peers and toys, and 10 min of free play with two guinea pigs and peers. As expected, significant differences in social anxiety were reported between the TD and ASD cohorts, with participants with ASD scoring significantly higher on the SWQ on both the parent and teacher versions (*p* < 0.001), demonstrating that children with ASD were perceived as less social, confident, and calm than the TD children. No differences in self-rated emotions between the TD and ASD cohort were found. There were differences between the conditions with all children feeling best in the presence of animals compared with toys, reading silently and reading aloud (*p* < 0.001). The presence of animals also resulted in decreased skin conductance in both TD and ASD cohorts. Interestingly, the ASD children displayed greater physiological arousal than TD peers in all conditions, but this trend reversed in the animal condition: animals reduced the general level of arousal and the number of skin conductance peaks in children with ASD compared to the TD children. Conclusions highlight a regulation of physiological stress and a stress buffering effect of the animal on both typically developing and children with ASD.

Lastly, Becker [43] specifically looked at the effect of physiological stress responses on executive functioning in the presence of either a real or toy dog. The study involved 38 children aged 8;0–14;6 years (N = 34 male, N = 4 female), and two dogs approved by school officials were employed. All children attended a special education school and had a formal diagnosis relating to behaviour, PDD, mood, anxiety, motor, psychotic, or some other, unspecified disorder. Children were assigned to either a real or toy dog condition, blood pressure and heart rate were monitored before and after testing in each condition, and participants were tested on three executive functioning tasks (coding, memory, and inhibition) using standardised tests: the Wechsler Intelligence Scale for Children, Fourth Edition (WISC-IV), the Picture Memory subtest from the Wide Range Assessment of Memory and Learning, Second Edition (WRAML-2), and the Inhibition subtest from the NEPSY-II (2007). Blood pressure and heart rate were measured using the Welch Allyn Spot Vital Signs [61], a device that provided digital measures of non-invasive pressure and pulse rate (heart rate). Results revealed that the presence of a dog had a significant effect on completion speed of the inhibition-naming task (F(1, 35) = 6.13, *p* = 0.018). However, no significant effect was revealed for Picture Memory, Inhibition-Naming, or Inhibition–Inhibition scores. Equally, none of the physiological variables of blood pressure and heart rate significantly predicted performance in executive functioning tasks. The study revealed a reduction in physiological arousal had occurred across the length of the intervention, but this was irrespective of whether the toy or real dog was present. As highlighted above, control groups should be included. In addition to investigating what the most advantageous length and timescales for interventions are, it is also important to look at these in relation to how novelty effects may impact intervention in the short term and whether any effects found can demonstrate longevity.

### 3.5. Motor Skills and Adherence to Instructions during Motor Tasks

Two studies in the review focused on motor tasks in the presence of a dog, Gee et al. [48] examined speed and accuracy of a set of gross motor tasks, whilst Gee et al. [24] investigated pre-schooler’s adherence to instructions when carrying out motor tasks. Both studies involved familiarised children with the dogs and used pre-school aged children; Gee [48] tested 4–6-year-olds (N = 14; N = 5 typical, N = 9 identified), and Gee at el. (2009) involved 3–5-year-olds (N = 11; N = 5 typical, N = 6 identified). Both studies use the term ‘identified’ to describe children who have a language impairment, and both involved intervention with a dog.

Gee [48] asked children to perform a series of 10 motor tasks either in the presence or absence of a therapy dog. A main effect of the presence of a dog was found (*F*(1, 36) = 7.471, *p* < 0.05, *R*^2^ = 0.17); children in the dog condition completed the task faster (*M* = 10.88 s) than those without a dog (*M* = 13.86 s). The type of motor task being carried out was also significant (*F*(5, 36) = 8.133, *p* < 0.05, *R*^2^ = 0.31), with some tasks being more adeptly performed in the presence of dog. The authors concluded that the presence of a therapy dog can be beneficial in the execution of gross motor skills and that the dog may act as motivator of performance in children. 

Gee et al. [24] used a motor task experiment to determine children’s ability to follow instructions. Children would perform the task in one of four co-performer conditions: with a real dog, a stuffed dog, a human confederate, or no co-performer. Each child had three types of motor tasks to complete: a ‘Modelling task’ in which the co-performer performed the task first whilst the child watched and then copied, a ‘Tandem task’ in which the child performed the task at the same time as the co-performer, and a ‘Competition task’ in which the child competed against the co-performer. 

The authors found a significant main effect of co-performer with children adhering to instructions better in the real dog and human confederate conditions than with the stuffed dog or when no co-performer was present (*p* < 0.01, *R*^2^ = 0.05). Task type and co-performer interaction also presented significant differences (*p* < 0.01, *R*^2^ = 0.19) with pairwise comparisons revealing that the Competition task presented no significant differences regardless of co-performer (*p* > 0.05). In the Tandem task, children adhered to instructions better in the presence of a human or stuffed animal (*p* < 0.05) whilst instructions were adhered to better in the Modelling task in the presence of a dog. 

Both studies presented the beneficial effects of interventions with animals during the execution of motor tasks. Discussion for further research focused on the small sample sizes used within the studies and the need to be able to look more in-depth at whether the presence of the dog showed more pronounced effects within either the typically developing children or the group of children with identified needs.

### 3.6. Adherence to Instructions and Memory Tasks

Gee et al. [25] explored the effect of a real dog, a stuffed dog, and a human confederate on the adherence to instructions during a memory task. The study involved pre-schoolers of 3–5 years of age (N = 12; N = 7 identified, N = 5 typical). The term ‘identified’ describes children who have learning deficits, behaviour deficits, or under-developed social skills. The real dog was a certified therapy dog, and the study was carried out in two parts. Experiment 1: Children took part in a forced choice recognition task in the presence of a co-performer: a real dog, a stuffed dog, or a human. Accuracy of choices and the number and type of instructional prompts required to carry out the task were measured. Task accuracy was at ceiling levels, with all children recognising objects with high levels of accuracy (*p* > 0.05). Analysis of general prompts revealed a significant difference between the real dog versus human conditions (*p* < 0.05), with the fewest required in the real dog (M = 1.17) as opposed to the human condition (M = 3.83). Pairwise comparison of task specific prompts also revealed a significant effect of these conditions (*p* = < 0.05), with children in the real dog (M = 0.83) and stuffed dog (M = 1.75) conditions requiring fewer prompts than did children in the human condition (M = 2.88) (*p* < 0.05). 

Experiment 2 was conducted six months later with the same children in order to replicate the first study. The study produced highly similar findings and confirmed the reduced need for instructional prompts in the presence of a real dog when completing cognitive memory tasks in the form of picture and object recognition. Hence, the study demonstrated positive results of the dog intervention. The authors discounted the effect of novelty as a reason for the positive effect of the dog due to the familiarisation process they provided, but ask whether the bonding process between child and dog drives the motivation of the child.

### 3.7. Categorisation and Object Recognition

Three of the papers from the Review investigated the effect of the presence of a dog on the categorisation and object recognition abilities of pre-school children [26,27,28]. All studies were carried out between a pre-school and lab setting, and testing sessions were single events. Dogs and handlers visited the educational setting on multiple occasions for familiarisation sessions before testing began so that the presence of a dog did not produce a novel situation for the children. The studies involved typically developing and identified pre-schoolers, with the authors defining the ‘identified’ cohort as children who have one or more difficulties in the following areas: oral expression, basic reading skills, listening comprehension, and written expression.

In addition, Gee et al. [27] tested 35–66-month-old pre-schoolers (N = 20; N = 12 typical and N = 8 identified) in their study looking at object recognition performance in the presence of a dog; object recognition involves both cognition and memory processes. Speed and accuracy across performance was measured whilst the number of distractor items and collaborator (dog versus humans) varied. Target and distractor items were purposely made similar, thus increasing the difficulty of the task being undertaken by the children. A main effect of number of distractors (*p* < 0.01) was found, with performance higher in the 1 distractor condition vs. 4 distractors. A significant effect of latency was revealed with children taking longer to respond in the 4-distractor condition (*p* < 0.01) and a significant effect of collaborator was also evident for both accuracy and latency such that performance was best in the dog condition. 

Similarly, Gee et al. [28] included 38–62-month-old pre-schoolers (N = 17; N = 11 typical and N = 6 identified) and showed that children also respond to exemplars more accurately in the presence of a dog (*p* < 0.01). Interestingly, there was a significant effect for animate versus inanimate classification, but this was only revealed in the real dog condition. 

Gee et al. [26] tested 36–63-month-old pre-schoolers (N = 12; N = 5 identified and N = 7 typical) and looked at the type of choices made by infants in relation to object categorisation in the presence of either a real dog, a stuffed dog, or a human confederate as a co-performer. Infants were required to take part in a ‘match-to-sample’ task with three categories of items to choose from: taxonomic, thematic, or irrelevantly linked items. As predicted, the children made significantly fewer irrelevant choices in the presence of a real dog (*M* = 0.58) (*p* < 0.05) than with the human (*M* = 1.0) or stuffed dog (*M* = 2.08). The results of the study also conformed to an expected developmental shift such that younger children made more taxonomic than thematic choices during object categorisation, whereas older children made more thematic than taxonomic choices. The study also revealed differences in the type of categorisation choices being made (*p* < 0.05) with taxonomic choices (*M* = 22.33) and thematic choices (*M* = 22.08) being made significantly more often than irrelevant choices (*M* = 3.67), demonstrating that children made relevant decisions in relation to the stimuli presented. The authors asserted a positive effect of the dog on categorisation performance. Consistent with the other studies in this review, the studies focusing on categorisation and object recognition also reported the positive effects of dog intervention. The authors referred to the need for arousal levels to be measured during future interventions to fully understand the special status of the dog, as opposed to the stuffed animal or human confederate.

The studies reported above, by Gee and colleagues, all relied on relatively small sample sizes, and thus lack broad generalizability. It is important to point out that they also implemented repeated measures designs and reported moderate to large effect sizes. Repeated measures designs offer the advantage of reducing error variance by allowing each participant to serve as his/her own control across experimental conditions. Additionally, these studies all involve an important familiarisation period where participants become acquainted with the dog and thus a novelty effect explanation cannot account for the findings.

### 3.8. Effect on Classroom Behaviour

Three papers within the review investigated the presence of an animal on classroom behaviour in general and with typically developing children. Two of the papers investigated the effect of the presence of a dog in the classroom [49,52], whilst the third involved a rabbit in the classroom environment [54]. The research by Kotrschal and Ortbauer [52] and Hergovich et al. [49] both included children in classes with a multi-ethnic background aged 6–7 years. Kotrschal and Ortbauer [52] familiarised the children with three dogs, two of which were certified therapy dogs. They video-recorded the class for a month before dog intervention and used this as a control to compare to videos of behaviour of the children in the presence of one dog during the school days in the intervention period. Video footage was coded for frequency of occurrence and duration across a large range of behaviours and was subsequently subjected to statistical analysis. However, the authors failed to specify the inter-rater reliability to ensure consistency of coding across conditions. 

Kotrschal et al. [52] found improved behaviour and, interestingly, more attention focused on the teacher with the dog present. They also reported previously withdrawn pupils as becoming more socially interactive. 

Hergovich et al. [49] also tested a class of children with a dog present after prior familiarisation and used a parallel class of children without a dog present as their control group. Assignment of classrooms to each condition was not random as the dog belonged to the class teacher (p. 41), a potential for bias. Hergovich et al.’s [49] measures included a selection of standardised tests, e.g., the Gestalt Perception Test (a measure of independence), the Vienna Development Test (social intelligence), and a self-assessment of empathy with animals [62], as well as teacher assessments of pupils’ sociability.

Hergovich et al. [49], as well as Kotrschal and Ortbauer [52], reported a more homogeneous classroom setting with a decrease in behavioural extremes such as aggression and hyperactivity. The rating of pupil’s sociability, social integration, and aggressive behaviour by Hergovich et al. [49] was carried out through a teacher assessment showing improvements, but this assessment is open to potential expectancy bias as teachers were not blind to conditions. Hergovich et al. [49] also found significant increases in empathy and field independence, but no differences in social intelligence between test and control groups.

Loukaki and Koukoutsakis [54] evaluated the effect of a rabbit in healthy pupils (N = 39) aged 2; 6–4 years within a pre-school classroom environment. Children were exposed to a rabbit twice per week for two hours within the classroom. The children could pet and care for the rabbit, in addition to educational activities also being planned around the animal. The authors concluded that the pupil’s ability of socializing, communicating, and expressing emotions increased significantly; however, whilst the study collected data on socialisation, communication, and emotional expression, these do not appear to be standardised measures. The lack of procedural detail and control conditions makes the study impossible to replicate and equally impossible to assess any beneficial effects gained through exposure to the rabbit. Additionally, the authors appear to present the rabbit as a commodity, with little appreciation of promoting animal welfare to children within classroom settings. Whilst it is important to protect the emotional welfare of children, the authors’ view that ‘rabbits can be practically “immortal” as they can be replaced with another individual of similar size and colour’, lacks respect for the animal and does not promote teaching children respect for animal welfare.

Again, all studies reviewed here reported beneficial effects of having an animal in the classroom setting, with a need for randomised controls with longitudinal assessment of the effects being raised as issues. It is also important to emphasise the importance of appropriately trained dogs and consideration of the legal, ethical, risk, and welfare implications when carrying out studies with animals.

## 4. Discussion

The current review assessed the extent and variability of research involving animal-assisted intervention to aid student behaviour and learning in the classroom setting, including effects on cognition, social and emotional functioning, motor skills, and physiological arousal. Most, but not all studies, concluded a beneficial effect of interactions with an animal. 

It is worth noting that, as most of the articles lacking significant beneficial effects originated through grey literature databases, this could represent a positive publication bias. All papers within the review were of empirical studies, and ultimately grey literature has provided this review with a more balanced view of animal-assisted interventions previously carried out in schools. Publication bias has implications for many scientific fields of study, including the broader field of human–animal interaction.

Studies within the review consisted of single case designs with the smallest cohort consisting of two participants [51], whilst the largest cohort consisted of 230 children in randomised controlled trials [57]. Eight of the studies involved pre-school children from the age of 2;6 years and upwards [24,25,26,27,28,47,48,54], 16 studies involved school-aged children up to 17;5 years of age, and the remaining study spanned across both [50]. Apart from three, all the studies included a mix of both genders. Only male children were recruited in two studies to reduce variance in the sample [44,45], and in a further study due to the case study design and the availability of suitable participants [51]. One study in the review included gender as a factor in much of their final analysis, but failed to state the gender split in their cohort [53].

In addition to typically developing children, the review highlights that children with a wide range of differing characteristics, including behavioural and learning difficulties, have been involved in research assessing the effect of animal-assisted intervention. These include Autism Spectrum Disorder [55,56], insecure/disorganised attached children [44,45], emotional/behavioural difficulties [41,42,51,59], children with identified deficits and/or difficulties with learning, language, and communication [24,25,26,27,28,43,48,53], visual processing challenges, auditory processing challenges and attention focus challenges [58]. One study [50] included a range of pupils from kindergarten to Grade 4, some in special education classes, but failed to specify learning deficits or numbers involved. Two studies also worked with children with a multi-ethnic background [49,52].

It is useful to note that, whilst the studies reviewed here took place in educational settings, the focus was not necessarily on educational/cognitive effects of interaction with animals. Four studies focused on social functioning [55,56,57,59], four on emotional stability [41,46,47,51], and three investigated physiological arousals during AAI sessions with children [43,44,45,56]. Three further studies looked at the effect of an animal on the classroom environment in general [49,52,54].

Whilst most papers within the review reported beneficial findings from their interventions with animals, it is evident that further research is needed to extricate and quantify the wide variety of factors involved across the findings. To do this successfully, one important element in this scenario is the design of the research project. Strict methodological protocols are desirable to carry out interventions as planned and are helpful when multiple measures are employed. Furthermore, it is important to learn how measures may interact with each other.

In the future, the incorporation of a higher quantity of randomised controlled trials with appropriate control groups would aid in attributing factors directly to the intervention implemented during animal-assisted activities. Control groups are an important element in research design, but they must be appropriate and serve their purpose effectively. Inappropriate control conditions can fundamentally flaw research outcomes, hindering the ability to determine effectiveness of experimental interventions. In this review, five of the studies integrated an independent control group into their design [46,47,49,58,59]. Four of the studies used randomised controlled designs with children allocated to different conditions, but none of these had a strictly separate control group of children without intervention for comparison [43,44,45,53,57]. Ten studies used the child as their own control [24,25,26,27,28,44,45,48,52]. Of the five remaining studies, three did not include a control group as they used case study designs with between 2 and 6 participants [41,42,51]. Two studies under review failed to include an appropriate control group to compare their sample against [50,54], leaving their results open to interpretation.

Even where controls are implemented, it is vitally important that wider factors, such as the effect of a teacher or school when using a whole class cohort, are controlled to reduce bias in results [46,47,59]. These confounding factors represent a design flaw and are a threat to internal validity. Some authors acknowledged the potential effects of the classroom or the school [56].

The type of design also has consequences for the interpretation of results. For example, repeated measures designs, compared to a between-subjects design, have the advantage of each child serving as his/her own control in the study. These designs allow one to examine the dependent measures before and after the animal is present, or to separate out the impact of the animal, from that of a toy version of the animal, from the absence of the animal. It is common for intervention studies to involve small sample sizes, and repeated measures designs provide for the collection of a large number of data points, with each subject serving as their own control (reduces error variance), which is a substantial advantage over a between-subjects design of the same sample size. 

It is also important that measures are applied equally. Treat [58] failed to carry out the main pre–post measurement of reading in the control group condition; while the intervention group demonstrated significant improvement on the task, the study failed to demonstrate the specific effect of the dog, as opposed to the individual researcher/teacher also present in each session. 

Further considerations linked to the design of the study concern the length of animal intervention sessions, longitudinal timescales (if any), and the type of animal contact involved. Analysis of the papers in this review revealed a variety of intervention setups and a distinct lack of consistency across the studies. Of the 25 studies, 9 involved single intervention sessions with a dog present during an experimental task [24,25,26,27,28,43,44,45,48]. The remaining studies involved longitudinal intervention between 1 and 12 months, with most interventions lasting between 2 and 3 months [41,47,49,50,51,52,53,54,55,56,57,58,59]. Within these time frames there was also wide variety in session duration, with exposure to animals ranging from 20 min per week [53,58] to the animal being present in class for the full school day or week [41,49,52,55,56]. One study varied the intervention duration within the study [59].

The type of interaction taking place was also reported in all the studies reviewed although there is generally not enough information included for complete replication. For example, information may be missing or unclear as to whether the child was seated next to the animal, having direct contact by grooming or petting, or whether the animal was exposed to all children in the classroom. Surprisingly, one study included a child with allergies participating in the study using an iPad and thus had no direct contact with a dog at all [50]. Direct contact is a particularly important factor as it may have an impact on the strength and longevity of effects and has been shown to affect physiological processes [17]. 

Only five of the studies reviewed included video analysis of the dog–child interactions taking place [41,44,45,51,52]. These studies did associate a deeper bonding process with, for example, increased social and co-operative behaviours in the classroom setting. Further analysis of these interactions could potentially answer the question of whether children will habituate to the presence of a dog over time, and at what point if any, the intervention may cease to be effective. 

Further questions arise, for example, if the quality of the human–animal relationship makes a difference to the sustainability of the intervention over time. Collecting measures in relation to pet ownership history and a child’s attachment to their pet will also help to ascertain whether these factors are likely to impact on the immediate outcomes of intervention and whether they make a difference to the sustainability of effects over time. 

Familiarisation with the intervention animal was carried out in some of the studies in order to negate any potential novelty effects of the animal in the pre-school setting but was not used as often in other studies within the review [24,25,26,27,28,48]. It would be useful to understand more fully what processes of familiarisation are required to counteract novelty effects, and whether there is a trade-off between familiarity and novelty effects in terms of the effectiveness and longevity of the interventions taking place. Furthermore, the familiarisation process and any intervention is likely to lead to bonding between child and dog, which in turn can alter the quality of the intervention. This warrants future investigations.

Familiarisation not only has the potential to counteract novelty effects, but also provides an opportunity to ensure that children and staff are trained in understanding stress-signalling behaviour of the animals. This is particularly important when considering the inclusion of dogs in the classroom and the potential risk to both parties involved, as a dog’s behavioural signals are often misinterpreted by both children and adults [63]. Hence, it is important that interventions involving dogs in schools and other educational settings are carried out using trained therapy dogs with trained handlers in attendance.

In the current review, all but three of the studies with dogs used trained or certified therapy or school dogs. Donaldson [47] involved PAWS for People dogs and handlers in their study and highlights the robust process for inclusion; dogs endorsed by the PAWS organisation are required to pass four sections of the Standards of Excellence (STEX) evaluation. PAWS will fully endorse, and certify, a therapy-dog team only after all areas are successfully completed. In addition, all therapy-dog teams must be re-evaluated every two years to maintain their certification. 

In contrast, Anderson [41] reported that the dog was not trained to interact for the intervention and had not previously experienced younger children. However, Anderson reported the strictest protocol for ensuring safety in the classroom by applying a four-part action plan to reduce risks to children and the dog. This included teaching the children about safe behaviours with dogs, for example, not to touch the dog whilst the dog ate or slept. Two other studies also included this beneficial step [49,52]. 

One study employed a certified dog handler/trainer, but the dogs were pets of other local community members and did not belong to the handler [59]. Worryingly, this study did not involve merely the dog being present in the room, but the students directly trained and interacted with the dog.

Only five of the studies mentioned collecting information on allergies [42,46,50,53], but six other studies by Gee and co-authors also included this (personal communication). While it is not stated explicitly within the methodology of the remaining papers reviewed, it is assumed that all the research involving animals would have included allergies as a factor on parental consent forms. Failure to do so would represent a considerable and serious omission. 

It is vital that legal, ethical and risk implications must be assessed during design and implementation of research. The welfare needs of children and school staff are paramount in such research, but equally, it is crucially important that the welfare needs of the dog are also carefully assessed concerning type of contact and the length of sessions involving direct contact time with the children [4].

Physiological measures of wellbeing are used as more direct measures that reflect stress and wellbeing in various contexts, including relationships and the bonding process. However, of the 20 papers under review, only four included the assessment of physiological measures; two measured skin conductance; two analysed cortisol [15,43,44,45]. These studies investigated the benefits of animals as social buffers in the classroom. Surprisingly, none of the remaining 16 studies took measures of physiological data to form a better understanding of the dynamics involved in human–animal interactions in the classroom. The collection of such measures would supplement researchers’ understanding of the effect of animals on children’s educational attainment.

This is not to say that self-reported measures of social and emotional wellbeing, anxiety, and stress are not appropriate. Indeed, checklists or feedback from those taking part may not be a direct measure of arousal or emotional condition, but can provide a detailed insight into the experiences of those involved in animal-assisted activities in schools such as that reported by Wicker [59]. Adding physiological data to the research could provide a richer understanding of the dynamics of human–animal interactions by combining a wide variety of both quantitative and qualitative measures for analysis. It would also provide a way to detect unconscious bodily reactions that escape humans’ awareness.

Kogan et al. [51] expressed concern that the two children in their sample received different goals during their intervention with dogs and pointed out the difficulty in controlling for external factors, especially in research with identified populations who may already be receiving additional services and interventions from other sources. Furthermore, it is important that more specific data such as the characteristics, ability, and special needs diagnoses of the child are gathered and included into future analyses. Different populations such as those diagnosed with ADHD or ASD often demonstrate differences in physiological reactivity to contact with animals, such as increases in heart rate or reductions in salivary cortisol, contrary to those observed in typically developing populations. The inclusion of physiological measures would allow for a more detailed examination of how individual differences impact, and moderate the effects of, animal-assisted interventions.

## 5. Conclusions

This review of animal-assisted intervention in educational settings demonstrates that the majority of the studies, though not all, reported beneficial effects on cognitive and socio-emotional behaviour and physiological responses. The review also highlights the large variation in the design of such studies and identifies multiple external factors that may influence results.

Among the factors that can make it hard to interpret the study results are sample selection and size—samples need to be appropriate in size and consistency, and so far sample sizes are often small and may, for example, contain mixed ages or mixed abilities. Many studies still fail to include an adequate control group into their design, reducing the ability to assess the effect of the intervention. 

Study designs and procedures could often benefit from more rigor, such as a random assignment to condition, the use of appropriate control conditions, the use of standardized measures, blind scoring of data, inter- and intra-rater reliability, and strict adherence to protocol. This would ensure that effects can be adequately assessed. 

We observed that the length of interventions, and hence exposure to the animals varied greatly across studies. It is not possible to analyse results of exposure here, but future research should focus on this point and assess timescales for optimal beneficial outcomes. It is furthermore important for researchers to describe the animals involved, the specifics of the interactions that take place during the study, participants’ previous experience with animals, and the degree to which the participants are attached to the animals in the study and their own pets. Overall, strategies to establish, monitor, and maintain accuracy and consistency of an intervention need to be in place and potential confounders noted. 

Thus, our recommendations on enabling more robust research in this field are as follows: it would be advisable if research studies were designed with rigor, containing appropriate sample size and selection, including adequate control groups. Testing should adhere to a strict protocol that is known to all involved, and study results should be reported with all necessary detail and background information so as to enable replication. Wherever possible, a range of measures should be employed, including standardized and physiological measures. Intervention studies require clear procedures for ensuring treatment fidelity. While case studies are valuable, they cannot replace randomized controlled trials. Animal welfare and safety precautions have to be taken into consideration and integrated into the research design and procedures.

In order to move the field forward additional studies are needed. So far, very few studies included the use of ethological measures. However, the number and type of direct interactions may also provide a deeper insight into the relationship between child and animal, demonstrating a trade-off between the quality and quantity of interactions and the resulting beneficial effects (including direct physiological outcomes). A deeper understanding of the bonding and attachment processes involved during child and animal interactions could be informed through ethological data, enabling researchers to better understand the beneficial effects and ultimately to clarify whether certain children may benefit from interactions more than others. 

Most of the studies involved dogs, it would be interesting to investigate further if different dog characteristics (e.g., breeds, temperaments, and sizes) and different types of animals influence the effects. Is a rabbit or guinea pig as effective as a dog? Does the impact of HAI depend on participants’ previous experience with different animal types? Hence, rigorously designed studies are needed that focus especially on the effects of duration and frequency of interventions, quality and quantity of interactions with different populations, as well as on the effects of different types of animals.

In addition, whilst it is crucial to ensure strict protocols for research design and procedure, it is also important that wider services and other interventions already provided by schools are not removed. In the future, it would be useful if these could be either integrated into research design or accounted for during analysis in order to demonstrate robust and applicable interventions in the face of complex requirements. Ideally, and given appropriate training and support, educational establishments may in future choose to use intervention flexibly and in innovative ways that could give children the best chances of success. Additionally, it would also be useful to establish the most cost-effective methods of intervention to educational settings. 

Finally, it is of crucial importance to assess whether including animals in educational settings is valuable or impactful. Do animals help children to learn? Do we see significant improvements in cognitive and socio-emotional behaviour outcomes and are these linked to changes in physiological states? It is vital that future research answers these questions and provides robust evidence as demonstrated through a child’s academic and socio-emotional outcome measures. In addition to beneficial outcomes for children, this would also ensure that unnecessary interactions are limited. This in turn may increase welfare considerations for the animals involved. In short, we need to determine the optimum and most effective course of intervention to provide the best outcomes for children.

Lastly, but importantly, research involving AAI needs to ensure a strict and thorough protocol for risk assessment measures such as the training level and certification of dogs/handlers, allergy and phobia information, and child safety training in relation to understanding dog behavioural signals; these will ultimately protect the welfare and safety of staff, children, and animals involved in interventions.

## Figures and Tables

**Table 1:** Oxford Centre for Evidence-Based Medicine (OCEBM) Levels of Evidence.

Level	Levels of Evidence	Qty of Articles
1	Systematic reviews of randomised trials or n − 1 trials	N/A
2	Randomised trial or observation study with dramatic effect	21
3	Non-randomised controlled cohort/follow-up study	0
4	Case series, case-control studies, or historically controlled studies	4
5	Mechanism-based reasoning	0

**Table:** (**a**)

First Author	OCEBM Rating	Participants				Type of Control Group within Study	Animal	Experimental Task During Intervention
		Group Size (N)	Age	Gender	Cohort Behavioural/Learning Difficulties in Addition to TD Cohort			
**Anderson 2006 [41]**	4	6	6–11 years	M = 3, F = 3	Children with severe emotional disorders; oppositional defiance disorder, attention deficit disorder with hyperactivity, reactive attachment disorder, intermittent explosive disorder, central auditory processing disorder, intermittent explosive disorder, Asperger’s syndrome	None: case study	Dog (not therapy)	Dog present in class, no task. Teacher presented half hour social instruction each morning
**Bassette 2013 [42]**	4	3	7–11 years	M = 2, F = 1	Emotional & behavioural difficulties	None: case study	Dog	Reading program
**Becker 2014 [43]**	2	38	8;0–14;6-years	M = 34, F = 4	Behavioural, PDD, Mood, Anxiety, Motor, Psychotic, learning disorder and Other disorder not specified.	None	Dog	Coding, inhibition & memory tasks = -WISC-IV Coding task -WRAML-2 Picture Memory subtest. -NEPSY-II Inhibition
**Beetz 2011 [44]**	2	31	7–12 years	M = 31, F = 0	Insecure-avoidant/disorganized attachment	Randomised control: Dog support, toy dog support & human support	Dog	Trier social anxiety test for children (TSST-C)
**Beetz 2012 [45]**	2	47	7–11 years	M = 47, F = 0	Insecure-avoidant/disorganized attachment	Randomised control: Dog support, toy dog support & human support	Dog	Trier social anxiety test for children (TSST-C)
**Beetz 2013 [46]**	2	46	8–9 years	M = 23, F = 23	None	Independent class control	Dog	None
**Donaldsonb 2016 [47]**	2	47	3;8–4;11 years	M = 23, F = 24	Developmental delay/disability (N = 4)	Independent class control	Dog	Emotional matching task
**Gee 2007 [48]**	2	14	4–6 years	M = 10, F = 4	‘Identified’ pre-schoolers having learning deficits, behaviour deficits, underdeveloped social skills as assessed by independent committee for preschool education	Each child took part in both conditions and acts as their own control	Dog	Gross motor skill tasks
**Gee 2009 [24]**	2	11	3–5 years	M = 8, F = 3	‘Identified’ pre-schoolers having learning deficits, behaviour deficits, underdeveloped social skills as assessed by independent committee for preschool education	Child acts as own control: Dog, stuffed dog, human & no co-performer	Dog	Motor skills
**Gee 2010a [25]**	2	12	3–5 years	M = 7, F = 5	‘Identified’ child has delays in the following areas, cognitive, speech & language or pragmatic skills	Child acts as own control: Dog, stuffed dog, human & no co-performer	Dog	Object categorisation task
**Gee 2010b [26]**	2	12	3–5 years	M = 6, F = 6	Identified’ child has one or more difficulties with oral expression, basic reading skills, listening comprehension, written expression.	Child acts as own control: Dog, stuffed dog, human & no co-performer	Dog	Memory Task
**Gee 2012a [27]**	2	20	2–5 years	M = 11, F = 9	‘Identified’ child has one or more difficulties with oral expression, basic reading skills, listening comprehension, written expression.	Child acts as own control: Dog collaborator, human collaborator	Dog	Object recognition performance
**Gee 2012b [28]**	2	17	3–5 years	M = 7, F = 10	‘Identified’ child has one or more difficulties with oral expression, basic reading skills, listening comprehension, written expression.	Child acts as own control: Dog collaborator, stuffed dog collaborator, human collaborator	Dog	Object categorisation
**Hergovich 2002 [49]**	2	46	6–7 years	M = 23, F = 23	Viennese Grade 1 classes at European School, Families of economic migrants	Control class without dog	Dog	None
**Kirnan 2016 [50]**	2	169	Kindergarten to 10 years	M = 85, F = 84	Students in traditional and special educational needs classrooms—not specified further. Mix of Caucasian, Asian, Hawaiian/Pacific Islander, Hispanic, and Other/Multi-racial.	MAP scores (reading) used as control for dog group in following year.	Dog	Reading task
**Kogan 1999 [51]**	4	2	11 & 12 years	M = 2	Child A: mild retardation, attention deficit disorder, oppositional defiant disorder, depression and explosive tendencies. Child B: hyperactive, depression & problems with impulse control. Both had emotional disorders.	None: case study	Dog	None
**Kotrschal 2003 [52]**	2	24	6–7 years	M = 14, F = 10	First generation immigrant families in mainstream class	Class acts as own control group—was video-taped for a month before intervention	Dog	None
**Le Roux 2014 [53]**	2	102	7–13 years	Not stated	Poor readers (as assessed by ESSI)	Randomised control: Dog, adult and teddy groups	Dog	Reading program
**Loukaki 2014 [54]**	4	39	2–5 years	Not stated	None	None	Rabbit	None
**O’Haire 2013 [55]**	2	128	5–13 years	M = 71, F = 57	Included ASD children but not analysed in paper	Child acts as own control	Guinea pigs	Classroom-based activities
**O’Haire 2014 [56]**	2	64	5–12 years	M = 50, F = 14	ASD	Child acts as own control	Guinea pigs	Classroom-based activities
**O’Haire 2015 [15]**	2	114	5–12 years	M = 60, F = 54	ASD	Child acts as own control: Reading silently (baseline control), scripted classroom activity-reading aloud, free play with peers/toys, free play with peers/toys and Guinea Pigs	Guinea pigs	None
**Tissen 2007 [57]**	2	230	7–10 years	M = 109, F = 121	None	Randomised control: Social training with dog, social training without dog & dog attendance without training.	Dog	Social training
**Treat 2013 [58]**	2	17	7–10 years	M = 11, F = 6	Identified learning disabilities included: Visual processing challenge, Auditory processing challenge, Attention focus challenge	Readers with teacher & dog, Readers with teacher and no dog	Dog	None
**Wicker 2005 [59]**	2	31	12;2–17;5 years	M = 22, F = 9	Emotional difficulty, Autistic-like behaviour	Control group without AAT; Individual, small group AAT	Dog	None

**Table:** (**b**)

First Author	Measures					Timing of Intervention Assessment			
	Cognitive	Motor	Behavioural	Physiological	Ethological	Baseline	Immediate Start/During	Immediately Following	Long Term Effects
**Anderson 2006 [41]**	None	None	Problem solving sheets and ABC Analysis Forms	None	Daily observations	Yes	No	Yes	Not assessed
**Bassette 2013 [42]**	STAR reading progress	None	Interviews	None	None	Yes	Yes	Yes	yes
**Becker 2014 [43]**	Subtests from: -WISC-IV -WRAML-2 -NEPSY-II	None	None	GSR—blood pressure and heart rate	None	No	Yes	No	Not assessed
**Beetz 2011 [44]**	None	None	Separation Anxiety Test (SAT); ‘My pet and I’—pet attachment questionnaire; The Trier Social Stress Test for Children (TSST-C); Self-Assessment of Stress (SAM)	Salivary Cortisol collection & analysis	Video behaviour of all sessions	Yes	Yes	Yes	Not assessed
**Beetz 2012 [45]**	None	None	Separation Anxiety Test (SAT); The Trier Social Stress Test for Children (TSST-C); Self-Assessment of Stress (SAM)	Salivary Cortisol collection & analysis	Video behaviour of all sessions	Yes	Yes	Yes	Not assessed
**Beetz 2013 [46]**	None	None	Depression scale for children (DKT); Emotional & Social experiences in school (FEESS 3–4); Emotional regulation in children and juveniles (FEEL-KJ); Personality questionnaire (NEO-FFI)	None	None	No	Yes	Yes	Not assessed
**Donaldson 2016 [47]**	None	None	Strengths and Difficulties Questionnaire (SDQ); Emotional Matching Task (EMT); Planned Activity Check (PLA-c); Challenging Situation Task (CST).	None	Video behaviour of certain sessions	Yes	Yes	Yes	Not assessed
**Gee 2007 [48]**	None	Experimental task	None	None	Video recorded	N/A	N/A	Yes	Not assessed
**Gee 2009 [24]**	None	Experimental task	None	None	Video recorded	N/A	N/A	Yes	Not assessed
**Gee 2010a [25]**	Experimental task	None	None	None	Video recorded	N/A	N/A	Yes	Not assessed
**Gee 2010b [26]**	Experimental task	None	None	None	Video recorded	N/A	N/A	Yes	Not assessed
**Gee 2012a [27]**	Experimental task	None	None	None	Video recorded	N/A	N/A	Yes	Not assessed
**Gee 2012b [28]**	Experimental task	None	None	None	Video recorded	N/A	N/A	Yes	Not assessed
**Hergovich 2002 [49]**	Coloured Progressive Matrices (CPM); Gestalt Perception Test (GWT); Vienna Development Test (WET)	None	Teachers’ assessments of pupil’s sociability, social integration & aggressive behaviour; Self-assessment of empathy with animals (as Killian, 1994)	None	None	Yes	Yes	No	Not assessed
**Kirnan 2016 [50]**	None	None	School MAP scores	None	None	No	No	Yes	Not assessed
**Kogan 1999 [51]**	None	None	Comprehensive Teacher Rating Scale (ADD-H); Personal IEPs; Practitioner post intervention interviews	None	Video: Direct observation & daily coded video	Yes	N/A	Yes	Not assessed
**Kotrschal 2003 [52]**	None	None	None	None	Video recorded	Yes	Yes	No	Not assessed
**Le Roux 2014 [53]**	Neal analysis of reading ability (1999)	None	None	None	None	Yes	No	Yes	Yes
**Loukaki 2014 [54]**	None	None	In-house questions on socialisation, communication & emotional expression	None	None	none stated	none stated	none stated	None stated
**O’Haire 2013 [55]**	None	None	Social Skills Rating System (SSRS)—subsets Social skills, Problem behaviours & Academic competence	None	None	Yes	No	Yes	Not assessed
**O’Haire 2014 [56]**	None	None	Pervasive Developmental Disorder Behaviour Inventory (PDDBI); Social Skills Rating Scale (SSRS)	None	None	Yes	No	Yes	Not assessed
O’Haire 2015 [15]	None	None	Social Communication Questionnaire (SCQ); Social Skills Rating System (SSRS); Social Worries Questionnaire (SWQ); Character description by parent & teacher; Emotional valence–child-rated	Skin conductance measures, incl. temperature	None	Yes	Yes	No	Not assessed
Tissen 2007 [57]	None	None	Assessment Aids for Teachers, (Janowski, 1981); Inventory for the Assessment of Impulsivity, Risk behaviour and Empathy (IVE); Bully/Victim-Questionnaire (Olweus, 1989)	None	None	Yes	Yes	Yes	Yes
Treat 2013 [58]	Gray Oral Reading Test (GORT-4); Basic Reading Inventory (BRI)	None	Reader Self-Perception Scale (RSPC); Reading Anxiety Scale and Parent Questionnaire (created in-house)	None	None	Yes	No	Yes	Not assessed
Wicker 2005 [59]	None	None	Behaviour Assessment System for Children’s Teacher Rating Scale (TRS-A); Behaviour Assessment System for Children’s Self-Report of Personality (SRP-A)	None	None	Yes	No	Yes	Not assessed

**Table:** (**c**)

First Author	Animal Welfare	Type of Contact	Approximate Length of Contact		Main Outcomes	Training Level of Animal	Risk and Ethical Considerations
	Animal Contact Hours (Length and Total Hours)		Weekly	Monthly	+/−/NE/Mixed (+ for Positive, − for Negative Findings; NE for No Effect; Mixed for Mixed Effects)		
**Anderson 2006 [41]**	8 a.m.–3 p.m. per day for 8 weeks (except 1 day for illness; 2 months = 240 h	Dog in class	30 h week	× 8 = 240 h	+ Positive emotional effects on children; Positive impact on learning respect, responsibility and empathy.	Not trained therapy dog Not trained to interact for the intervention. Dog had not previously experienced young children	Ethics board. Protocol for ensuring safety in classroom whilst dog roaming. Allergies to dogs thro parent interview Children taught safe contact behaviours i.e., not touching dog whilst it ate, slept etc.
**Bassette 2013 [42]**	30 min per day × 4 weeks; 1 month = 10 h	Dog sat next to child	2.5 h week	× 4 = 10 h	+ Increased engagement and on-task behaviour during reading	Trained therapy dogs	Ethics board. Children included who were not fearful or allergic to dogs
**Becker 2014 [43]**	30 min per child in room whilst doing experimental task	25 min dog in room 3 min direct interaction		one-off support	Mixed Significant effect on executive function performance; No effect on physiological stress.	Not trained therapy dog. Owned by school teacher	Approved by university review board and school approved. Consent & allergy information from parent. Children were excluded based on school assessment of inability to interact appropriately with animals (behavioural school)
**Beetz 2011 [44]**	25 min per child during a full day	Free interaction with child as social support	25 min	one-off support	Mixed No effect of Self-reported stress levels between groups; Significant effect of dog on salivary cortisol.	Trained therapy dogs	None stated
**Beetz 2012 [45]**	25 min per child during a full day	Free interaction with child as social support	25 min	one-off support	Mixed No effect of Self-reported stress levels between groups; Significant effect of dog on salivary cortisol	Trained therapy dogs or school-dog	None stated
**Beetz 2013 [46]**	1 day per week, over full school year	1 day per week free roaming	6 h week		Mixed Significant improvement in positive attitude towards school and emotions relating to learning; No significant effect in relation to depression scores	Experienced school-dog	Ethics board Absence of allergies in the class
**Donaldson 2016 [47]**	9.50–11.10 2 mornings per week over 9 weeks; up to 21 h	In enrichment area of class–interaction	35 min per child per week	2 h 20 min	Mixed No improvement in emotion recognition; No improvement in prosocial, aggressive or isolation behaviours; Mixed results in relation to challenging behaviours.	Certified therapy dogs—fully assessed	Ethics and consent in appendices
**Gee 2007 [48]**	Dog had 15 min break within each half hour	Dog performed motor task with children	15 min	one-off support	+ Children completed tasks faster in presence of dog and with greater accuracy.	Certified therapy dogs	University Institutional Review Board
**Gee 2009 [24]**	2 dogs—each in school on alternate days. 1 in 4 tasks involved a dog; time for rest between	Performed motor task with or before child	15–20 min	one-off support	Mixed Presence of a dog had significant effect on children’s compliance with instructions in motor tasks requiring modelling; No effect of dog in tasks involving competition or tandem.	Certified therapy dogs	University Institutional Review Board
**Gee 2010a [25]**	30 min per day × 4 weeks	Sat with child		one-off support	+ Children made significantly fewer irrelevant choices in the presence of a dog.	Certified therapy dogs	University Institutional Review Board
**Gee 2010b [26]**	dog not present every day of testing	Dog present next to child		one-off support	+ Children require significantly fewer instruction prompts in presence of dog.	Certified therapy dogs	University Institutional Review Board
**Gee 2012a [27]**	60–90 min, twice per week	Sat with child	5 min per child-task	one-off support	+ Object recognition task performed significantly faster and more accurately in presence of dog.	Certified therapy dogs	Letter for consent sent home to parents; University Institutional Review Board
**Gee 2012b [28]**	60–90 min, twice per week	Sat with child	10 min per child -task	one-off support	+ Significant effect of dog on the categorisation of animate objects.	Certified therapy dogs	Letter for consent sent home to parents; University Institutional Review Board
**Hergovich 2002 [49]**	Dog present in class for 3 months from 8 a.m.–12 p.m.; 3 months = 240 h	Free roaming, children allowed to pet the dog	20 h week	8 h	Mixed Significant effect of dog on field independence and empathy; Non-significant effect on social intelligence.	Trained therapy dogs	Start of study, children taught how to care for a dog e.g., pet, feed, give a toy to dog
**Kirnan 2016 [50]**	1 h per class over 1 school year	Dog sat with group	1 h with group		Mixed Significant effect of dog on reading scores for Kindergarten only; No significant effect of dog on reading scores for children in Grades 1–4.	Trained therapy dogs	Schedule drawn up so dogs not overworked working with 5 classes for 1 h per week. Letter sent home to parents and parental permission obtained
**Kogan 1999 [51]**	Dog with child 45–60 min per week for 12 weeks; 9–12 h	General interaction and bonding alongside other tasks	45–60 min	3–4 h	+ All data sources report a significant improvement in individual goals.	Human–animal team	None stated
**Kotrschal 2003 [52]**	Dog present in class for one month, children videoed for 2 h, 3 times per week	Interact with dogs in a respectful manner at any time during their presence	All day	School for full day during one month	+ Significant effect on class socialisation, increased social integration and decrease in behavioural extremes.	Teacher owned, well-trained dog.	Worked with school to overcome bureaucratic hurdles. Boundaries set with children at start of project to instruct about dog’s needs, care & handling.
**Le Roux 2014 [53]**	Dog reading 20 min; 2.5 months = 3 h 20 min	Dog sat with child-child read to the	20 min per week	1 h 20 min	+ Significantly higher reading rate, accuracy and comprehension scores with a dog present;	Trained therapy dogs	Ethics board. None of children taking part were allergic to dogs
**Loukaki 2014 [54]**	In a transparent box in classroom twice per week for 2 h for 6 months; 96 h	General	4 h per week	16 h	+ Pupils’ socialising, communicating and expressing emotions increased significantly.	None mentioned	None stated
**O’Haire 2013 [55]**	5–6 h per day over 8 weeks	Responsibility for feeding, grooming and general care for the Guinea pigs.	25 h week	Guinea pig in class all day for 2 months	+ Greater improvements in social functioning and decrease in problem behaviours after intervention.	Consideration for type of animal partly based on child safety	Ethics board
**O’Haire 2014 [56]**	5–6 h per day over 8 weeks	Responsibility for feeding, grooming and general care for the Guinea pigs.	25 h week	Guinea pig in class all day for 2 months	+ Significant improvements in social approach behaviours & social skills, decreases in social withdrawal behaviours after intervention.	Consideration for type of animal partly based on child safety	Ethics board
**O’Haire 2015 [15]**	2 × 20 min sessions per week, over 8 weeks intervention; 2 months = 5 h 20 min	Free play	40 min	2 h 40 min	+ Significant reduction in heightened arousal when animal present; Significant decrease in skin conductance measures.	Consideration for type of animal partly based on child safety	None stated
**Tissen 2007 [57]**	90 min per week over 10 weeks; 2.5 months = 15 h	Children interacted with dog as part of the social task	90 min week	6 h	Mixed Significant improvement in pupils’ social behaviour and empathy overall; Non-significant effect of animal condition	Trained therapy dogs	None stated
**Treat 2013 [58]**	10–15 min varied 1–3 times per child, per week; 2–2 h 30 min max	Guided reading with dog and teacher/researcher	10–15 min × 3 30–45 min	Varied	+ Significant effect of dog on reading skill; Increase in feelings of self-efficacy, decreases in anxiety and increases in motivation to read.	Trained therapy dog	States certified therapy dog
**Wicker 2005 [59]**	1 to 1 = 1 h per week Group = 2 × 1 h per week 10 weeks; 10 weeks 1 to 1 = 10 h Group = 80 h	Training dog and learning about dog	1 to 1 = 1 h Group = 2 × 1 h	1 to 1–4 h Group–8 h	Mixed No significant effect of dog on social skills, aggressive behaviour, attitude to school, interpersonal relations or class absence; Anecdotal staff feedback reported positive impact on student behaviour and increasing self-confidence.	Trained dog handler Dogs from community members	Not certified dog parental permission sought
